# Novel *FGFR2*-*INA* fusion identified in two low-grade mixed neuronal-glial tumors drives oncogenesis via MAPK and PI3K/mTOR pathway activation

**DOI:** 10.1007/s00401-018-1864-5

**Published:** 2018-05-16

**Authors:** Payal Jain, Lea F. Surrey, Joshua Straka, Minjie Luo, Fumin Lin, Brian Harding, Adam C. Resnick, Phillip B. Storm, Anna Maria Buccoliero, Mariarita Santi, Marilyn M. Li, Angela J. Waanders

**Affiliations:** 1 Center for Data Driven Discovery in Biomedicine, Children’s Hospital of Philadelphia, 3501 Civic Center Boulevard, Philadelphia, PA 19104 USA; 2 Department of Pathology and Laboratory Medicine, Children’s Hospital of Philadelphia, Philadelphia, PA 19104 USA; 3 Department of Pathology and Laboratory Medicine, Perelman School of Medicine, University of Pennsylvania, Philadelphia, PA USA; 4 Department of Neurosurgery, Children’s Hospital of Philadelphia, Philadelphia, PA 19104 USA; 5 Meyer Children’s Hospital, Florence, Italy; 6 Department of Pediatrics, Perelman School of Medicine University of Pennsylvania, Philadelphia, PA 19104 USA; 7 Division of Oncology, Children’s Hospital of Philadelphia, Philadelphia, PA 19104 USA

As a group, mixed neuronal-glial tumors (MNGTs) exhibit genetic variability, including stable genomes, whole chromosome gains, *BRAF*-V600E, and *FGFR1* mutations [8, 9, 11, 12]. While histologic criteria are described to distinguish MNGT types ganglioglioma (GG) and dysembryoplastic neuroepithelial tumor (DNT), non-specific features preclude confident classification in a high proportion of cases [2, 8, 10, 12]. Herein, we report the characterization of a novel *FGFR2*-*INA* fusion gene identified during clinical genomic profiling in two cases of MNGTs that could not be specifically classified as GG or DNT.

Clinical, imaging, histology, and fusion gene characteristics of each case are summarized in suppl. Table 1 (Online Resource 1). Both patients presented with seizures, cortical-based tumors, and one patient’s tumor was recurrent. By histology and immunohistochemistry, both cases consisted of oligodendrocyte-like cells and admixed neurons within microcytic spaces (Fig. 1a). GFAP-positive astrocytes, CD34 expression (MNGT-1), and calcification were observed. Both cases lacked pools of mucin, floating neurons, specific glioneuronal elements, eosinophilic granular bodies, and perivascular inflammation. Features were most similar to DNT; however, both lacked key criteria for this diagnosis.

**Fig. 1:**
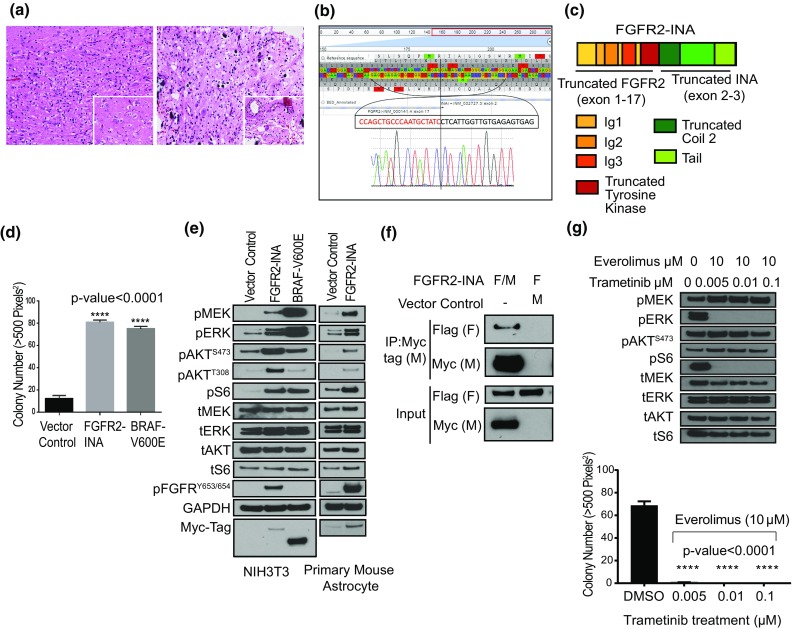
Histologic and sequencing characteristics of two MNGT harboring an *FGFR2*-*INA* fusion that activates the MAPK and PI3 K/mTOR pathways. **a** MNGT-1 (left) and MNGT-2 (right) contained small oligodendrocyte-like cells admixed with neurons surrounded by clear microcystic spaces (insets, 400X H&E), 200X H&E. **b** RNA-seq reads and confirmatory reverse complement Sanger sequencing of *FGFR2*-*INA*. **c** Structure of FGFR2-INA: *FGFR2* exons 2–3 encode Ig-1, exons 4–5 encode Ig-2, exons 6–7 encode Ig-3 domains, and exons 9–17 encode a truncated tyrosine kinase domain (lacking three amino acids from FGFR2 exon-18). **d** Soft agar assay using NIH3T3 stably expressing FGFR2-INA, n = 10. Error bars represent SEM. **e** Western blot analysis of MAPK and PI3 K/mTOR pathway proteins in NIH3T3 and PMAs. ‘p’—phosphorylated; ‘t’—total protein. **f** Co-immunoprecipitation (Co-IP) assay with anti-Myc tag beads and co-transfecting HEK293 cells with Flag (F)- and Myc (M)-tagged FGFR2-INA, and F-FGFR2-INA with M- vector control. **g** Effect of combinatorial trametinib and everolimus treatment on FGFR2-INA-driven oncogenic signaling and growth in NIH3T3 cells

Targeted RNA-sequencing revealed a novel in-frame fusion between *FGFR2* exon 17 and *INA* exon 2 (Fig. 1b) in both cases. Additional DNA sequence and copy number variants of clinical significance were also identified by targeted next-generation sequence panel [suppl. Tables 2, 3, 4 (Online Resource 1)] [7]. FGFR2, a receptor kinase, regulates several growth-related signaling pathways implicated in cancer progression, including RAS-RAF-MAPK and PI3K/AKT/mTOR [3]. INA encodes the alpha-internexin protein involved in cytoskeletal organization and neuronal morphogenesis [6]. The novel fusion retains the extracellular immunoglobin-like and tyrosine kinase domains of FGFR2, suggesting oncogenic activation of downstream signaling, and the truncated coil 2 and tail region of INA, suggesting dimerization (Fig. 1c).

We cloned *FGFR2*-*INA* and stably expressed it in NIH/3T3 and *Tp53*-null primary mouse astrocytes (PMAs) [1, 5] [suppl. Figure 1 (Online Resource 2)]. In soft agar proliferation assays, FGFR2-INA expressing NIH/3T3 showed a significant increase in colony count over control, similar to *BRAF* V600E (*p* < 0.0005) (Fig. 1d). Next, we assessed the signaling potential of FGFR2-INA. In serum starved conditions, we observed high-level activation of both the MAPK and PI3 K/mTOR pathways assessed via elevated levels of phosphorylated-ERK and -S6, respectively, compared to vector-controlled cells (Fig. 1e). Mechanistically, we found that FGFR2-INA homo-dimerizes in co-immunoprecipitation assays suggesting dimerization-induced activation of FGFR2-INA (Fig. 1f). Using combinatorial targeting of downstream MAPK and PI3K/mTOR pathways with trametinib and everolimus, respectively, we could suppress FGFR2-INA-driven oncogenic signaling and growth (Fig. 1f, suppl. Figure 2 (Online Resource 3)).

We identify and characterize a novel FGFR2-INA fusion associated with unclassified MNGT in two patients lacking other reported driver alterations (*BRAF*-V600E and *FGFR1*). Other *FGFR2* fusions have been identified in epileptogenic tumors of the young with some overlapping histologic features to the current two cases [4]. It is possible that these tumors represent an emerging category of low-grade epileptogenic tumor. Our functional studies show that the FGFR2-INA fusion drives oncogenesis potentially via activation of the MAPK and PI3 K/mTOR pathways. Therefore, FGFR2-INA is the likely driver of tumorigenesis in at least a subset of MNGTs and is a potential target for small-molecule inhibitors.

## Electronic supplementary material

Below is the link to the electronic supplementary material.

Supplementary material 1 (PDF 321 kb)

Supplementary material 2 (PDF 910 kb)

Supplementary material 3 (PDF 2077 kb)

Supplementary material 4 (PDF 50 kb)
