# Supplementary material for: Novel FGFR2-INA fusion identified in two low-grade mixed neuronal-glial tumors drives oncogenesis via MAPK and PI3K/mTOR pathway activation
Source: Acta Neuropathol. 2018 May 16;136(1):167–9. doi: 10.1007/s00401-018-1864-5 (PMC6015095; doi:10.1007/s00401-018-1864-5)
Supplement: Supplementary file 1 — Supplementary material 1 (PDF 321 kb) [file 401_2018_1864_MOESM1_ESM.pdf]

		MNGT-1	MNGT-2
**Age (years)**		6	9
**Gender**		M	F
**Location**		Temporal	Parietal
**Symptoms**		New onset seizures	Lifelong seizures, resolved after first surgery 5 years prior
**Imaging Characteristics**		Cortial based, small cystic areas, T2 hyperintense	T1 and T2 signal alteration extending from surgical cavity
**Primary vs. Recurrent**		Primary	Recurrent (after 5 years)
**Glial-Neuronal element (GNE)**		No	No
**Pools of Mucin**		No	No
**Oligodendroglial-like cells**		Yes	Yes
**Neurons**		Yes	Yes
**Floating Neurons**		No	No
**Astrocytes**		Yes	Yes
**Calcification**		Yes (sparse)	Yes (abundant)
**Microvascular changes**		No	No
**Rosenthal Fibers**		No	No
**Eosinophilic Granular Bodies**		No	No
**Necrosis**		No	No
**CD34 Immunohistochemistry**		Positive	Obscured by calcification
**Ki-67**		1-2%	<1%
**Gene Fusion**	**5' Partner**	FGFR2 (NM_000141.4) exon 17	FGFR2 (NM_000141.4) exon 17
	**3' Partner**	INA (NM_032727.3) exon 2	INA (NM_032727.3) exon 2

Supplemental Table 1. Summary of the clinical, radiographic, histopathologic, and *FGFR2-INA* fusion in MNGT.

MNGT #	Chromosome	Position^	TranscriptID	Nucleotide change	Gene	Expected Protein change	Classification*	VAF	Exonic Function	ExaC Frequency	dbSNP	SIFT (v6.2.0)	MutationTaster (v2013)	Nucleotide/Amino Acid Conservation	COSMIC Entry	Notes
1	chr 9	139410051	NM_017617.4	c.1787C>T	*NOTCH1*	p.Thr596Met	Tier 3	0.46	nonsynonymous SNV	0.0002	rs61755997	deleterious	disease causing	high/high	CM061876	
1	chr19	37866098	NM_004448.3	c.607C>T	*ERBB2*	p.Arg203Cys	Tier 3	0.44	nonsynonymous SNV	N/A	N/A	tolerated	polymorphism	none/weak	COSM3970014	
1	chr19	30308110	NM_001238.3	c.247G>C	*CCNE1*	p.Asp83His	Tier 3	0.44	nonsynonymous SNV	0.0003	rs147120845	tolerated	N/A	weak/moderate	No	
2	chr11	108155009	NM_000051.3	c.3802delG	*ATM*	p.V1268*	Pathogenic	0.53	stopgain	0.00003	rs587779834	N/A	N/A	N/A	COSM1315821	Confirmed germline on follow-up testing
2	chr1	118166196	NM_017709.3	c.706C>A	*FAM46C*	p.P236T	Tier 3	0.48	nonsynonymous SNV	N/A	N/A	deleterious	disease causing	high/high	No	
2	Chr1	120458982	NM_024408.3	c.6363G>C	*NOTCH2*	p.K2121N	Tier 3	0.46	nonsynonymous SNV	0.0004	rs144047610	tolerated	disease causing	weak/high	No	
2	chr1	161298206	NM_003001.3	c.98C>T	*SDHC*	p.T33M	Tier 3	0.40	nonsynonymous SNV	0.000057	rs148566767	tolerated	disease causing	moderate/moderate	No	

Supplemental Table 2. Single nucleotide variants identified in two MNGT tumors.

Abbreviation: VAF = Variant allele fraction, N/A = not applicable, SNV = single nucleotide variant
^Genomic position based on genome build hg19.
*Based on Li et al. 2017 JMD [7] except for ATM variant which was confirmed germline and is classified based on ACMG Criteria (Richards et al. 2015 PMID: 25741868)

Chromosome	Abnormality	Copies	Notes
X WC	Gain	4	
2 WC	cnLOH	2	
3 WC	Gain	3	
5 WC	Gain	3	
6 WC	Gain	3	
7 WC	Gain	4	includes BRAF
8 WC	Gain	3	includes FGFR1
9 WC	Gain	4	
10 WC	cnLOH	2	
11 WC	Gain	3	
12 WC	Gain	3	
13 WC	cnLOH	2	
14 WC	Gain	3	
15 WC	Gain	3	
16 WC	cnLOH	2	
19 WC	Gain	4	
20 WC	Gain	3	
22 WC	Gain	3	

Supplemental Table 3. Copy number variants identified in MNGT-2.
Note: No copy number changes were detected in MNGT-1
Abbreviations: WC- whole chromosome; cnLOH- copy neutral loss of heterozygosity

**RNA-seq panel included at least 1 or more targets in the following genes:**	**DNA-based NGS sequencing panel included the coding region and 5 base pairs flanking intronic sequences of the following 237 genes:**
ABL1, ABL2, AKT3, ALK, ARHGAP26, AXL, BCL2, BCL6, BCR, BRAF, BRD3, BRD4, CAMTA1, CBFB, CCNB3, CCND1, CIC, CRFL2, CSF1R, DUSP22, EGFR, EPC1, EPOR, ERG, ESR1, ESRRA, ETV1, ETV4, ETV5, ETV6, EWSR1, FGFR1, FGFR2, FGFR3, FGR, FOXO1, FUS, GLI1, GLIS2, HMGA2, IL2RB, INSR, JAK2, JAZF1, KMT2A, MALT1, MAML2, MAST1, MAST2, MEAF6, MECOM, MET, MKL1, MKL2, MSMB, MUSK, MTB, NCOA2, NOTCH1, NOTCH2, NRG1, NTRK1, NTRK2, NTRK3, NUMBL, NUP214, NUP98, NUT, PAX5, PDGFB, PDGFRA, PDGFRB, PICALM, PIK3CA, PKN1, PLAG1, PPARG, PRKCA, PRKCB, PTK2B, RAF1, RARA, RBM15, RELA, RET, ROS1, RSPO2, RSPO3, RUNX1, RUNX1T1, SS18, STAT6, TAF15, TAL1, TCF12, TCF3, TERT, TFE3, TFEB, TFG, THADA, TMPRSS2, TSLP, TYK2, USP6, YWHAE	*ABL1, ACVR1, AKT1, AKT2, AKT3, ALK,APC, AR, ARAF, ARID1A, ARID1B, ARID2, ASXL1, ATM, ATR, ATRX, AURKA, AURKB, AXIN1, AXL, B2M, BAP1, BARD1, BCL2, BCL6, BCOR, BCORL1, BLM, BRAF, BRCA1, BCRA2, BRD4, BRIP1, CARD11, CBFB, CBL, CCND1, CCND2, CCND3, CCNE1, CD274, CD79B, CDC73, CDH1, CDK12, CDK4, CDK6, CDK8, CDKN1B, CDKN2A, CDKN2B, CDKN2C, CHEK1, CHEK2, CIC, CREBBP, CRKL, CRLF2, CSF1R, CTCF, CTNNB1, DAXX, DDR2, DNMT3A, DOT1L, EED, EGFR, EP300, EPHA3, EPHA5, EPHB1, ERBB2, ERBB3, ERBB4, ERG, ESR1, ETV6, EZH2, FAM46C, FANCA, FANCC, FBXW7, FGF19, FGF3, FGF4, FGFR1, FGFR2, FGFR3, FGFR4, FLCN, FLT1, FLT3, FLT4, FOXL2, FOXP1, FUBP1, GATA1, GATA2, GATA3, GNA11, GNAQ, GNAS, GRIN2A, GSK3B, H3F3A, HGF, HIST1H1C, HIST1H3B, HNF1A, HRAS, IDH1, IDH2, IGF1R, IKBKE, IKZF1, IL7R, INPP4B, IRF4, IRS2, JAK1, JAK2, JAK3, JMJD1C, JUN, KDM5A, KDM5C, KDM6A, KDR, KEAP1, KIT, KMT2A, KMT2C, KRAS, MAP2K1, MAP2K2, MAP2K4, MAP3K1, MAPK1, MCL1, MDM2, MDM4, MED12, MEF2B, MEN1, MET, MITF, MLH1, MPL, MRE11A, MSH2, MSH6, MTOR, MUTYH, MYB, MYC, MYCN, MYD88, MYOD1, NF1, NF2, NFE2L2, NKX2-1, NOTCH1, NOTCH2, NPM1, NRAS, NTRK1, NTRK2, NTRK3, PALB2, PAX5, PBRM1, PDCD1, PDGFRA, PDGFRB, PHOX2B, PIK3CA, PIK3CG, PIK3R1, PIK3R2, PIM1, PPM1D, PPP2R1A, PRDM1, PRKAR1A, PTCH1, PTEN, PTPN11, RAD50, RAD51, RAF1, RARA, RB1, RET, RHOA, RICTOR, RNF43, ROS1, RPTOR, RUNX1, SDHA, SDHB, SDHC, SDHD, SETD2, SF3B1, SMAD2, SMAD4, SMARCA4, SMARCB1, SMO, SOCS1, SOX2, SPEN, SPOP, SRC, STAG2, STK11, SUFU, SUZ12, TERT, TET2, TGFBR2, TNFAIP3, TNFRSF14, TOP1, TP53, TP63, TSC1, TSC2, TSHR, U2AF1, VHL, WHSC1, WT1, AMER1, XPO1*

Supplemental Table 4. Targeted RNA-seq fusion panel genes and 237 cancer related genes sequenced by NGS

		FGFR2 e17 - *INA* e2
Primer Sequence	**Inner left**	CGAATTCTCACTCTCACAACCA
	**Inner right**	GGTGTTCCTCAGATCATTCTCC
	**Outer left**	GAAGGAAGGACACAGAATGGAT
	**Outer right**	TTTTGACATTGAGCAAGTCCTG

Supplemental Table 5. PCR Primers for *FGFR-INA* fusion confirmation
