# Supplementary figures and images for: Novel FGFR2-INA fusion identified in two low-grade mixed neuronal-glial tumors drives oncogenesis via MAPK and PI3K/mTOR pathway activation
Source: Acta Neuropathol. 2018 May 16;136(1):167–9. doi: 10.1007/s00401-018-1864-5 (PMC6015095; doi:10.1007/s00401-018-1864-5)

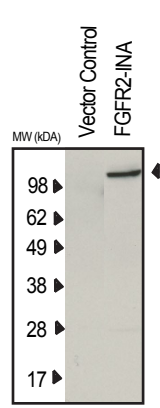

NIH3T3

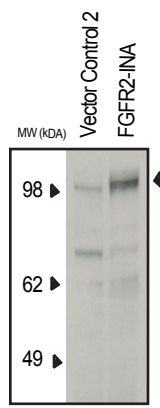

Primary Mouse Astrocytes

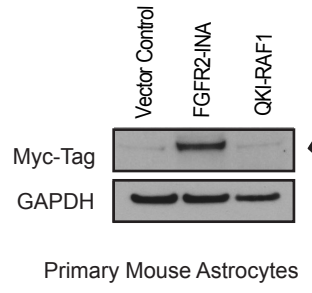

Supplement: Supplementary file 2 — Supplementary material 2 (PDF 910 kb) [file 401_2018_1864_MOESM2_ESM.pdf]

a.

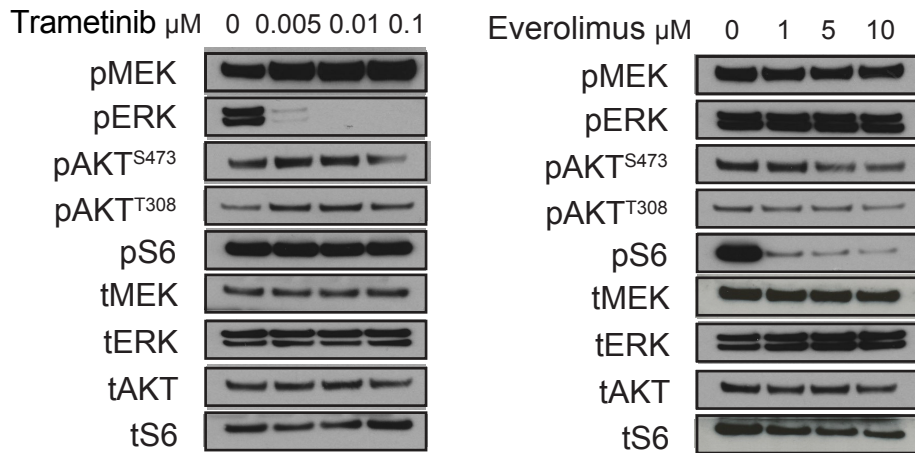

b.

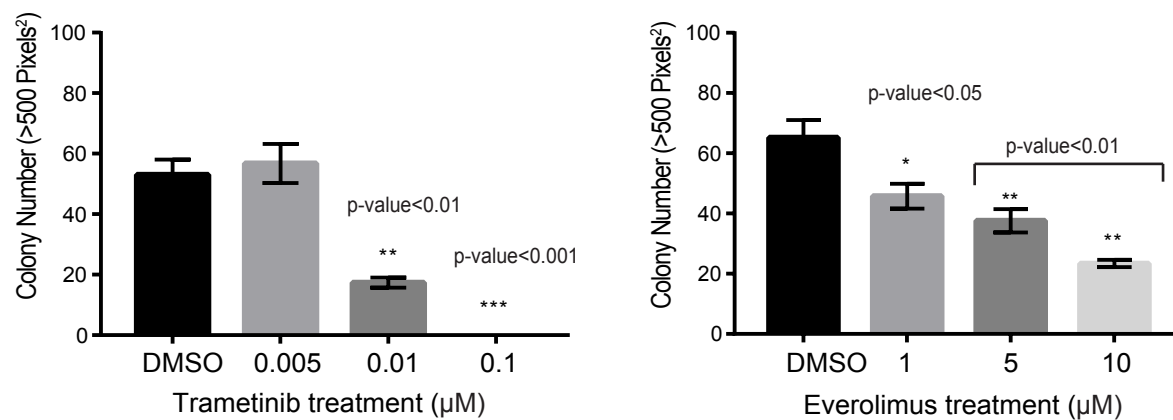

NIH3T3 expressing FGFR2-INA

Supplement: Supplementary file 3 — Supplementary material 3 (PDF 2077 kb) [file 401_2018_1864_MOESM3_ESM.pdf]
