# Supplementary material for: Novel FGFR2-INA fusion identified in two low-grade mixed neuronal-glial tumors drives oncogenesis via MAPK and PI3K/mTOR pathway activation
Source: Acta Neuropathol. 2018 May 16;136(1):167–9. doi: 10.1007/s00401-018-1864-5 (PMC6015095; doi:10.1007/s00401-018-1864-5)
Supplement: Supplementary file 4 — Supplementary material 4 (PDF 50 kb) [file 401_2018_1864_MOESM4_ESM.pdf]

SUPPLEMENTAL INFORMATION FOR:

Novel *FGFR2-INA* fusion identified in two low-grade mixed neuronal-glial tumors drives oncogenesis via MAPK and PI3K/mTOR pathway activation

Payal Jain[1]*, Lea F. Surrey[2,3]*, Joshua Straka[1], Minjie Luo[2,3], Fumin Lin[2], Brian Harding[2,3], Adam C Resnick[1], Phillip B Storm[4], Anna Maria Buccoliero[5], Mariarita Santi[2,3], Marilyn M. Li[1,2,3,6**], Angela J Waanders[1,6,7**]

1. Center for Data Driven Discovery in Biomedicine, Children's Hospital of Philadelphia, Philadelphia, PA, 19104, USA
2. Department of Pathology and Laboratory Medicine, Children's Hospital of Philadelphia, Philadelphia, PA, 19104, USA
3. Department of Pathology and Laboratory Medicine, Perelman School of Medicine, University of Pennsylvania, Philadelphia, Pennsylvania
4. Department of Neurosurgery, Children's Hospital of Philadelphia, Philadelphia, PA, 19104, USA
5. Meyer Children's Hospital, Florence, Italy
6. Department of Pediatrics, Perelman School of Medicine University of Pennsylvania, Philadelphia, PA 19104
7. Division of Oncology, Children's Hospital of Philadelphia, Philadelphia, PA 19104

*Co-first authors, contributed equally, **Co-last authors, contributed equally

Corresponding author:

Angela J. Waanders MD, MPH
CTRB 4030, 3501 Civic Center Boulevard, Philadelphia, PA 19104. Tel: 215-687-9550; Fax: 215-590-9956; Email: WAANDERSA@email.chop.edu

SUPPLEMENTAL METHODS

Immunohistochemistry. Immunohistochemical assessment was performed using antibodies against neurofilament NFP (RMd0-20, 1:400, Invitrogen, Carlsbad, CA), GFAP (6F2,1:400, Agilent, Wilmington, DE), CD34 (QBEnd-10, 1:50, Agilent, Wilmington, DE), Ki67 (MIB-1,1:200, Agilent, Wilmington, DE), NeuN (A60, 1:1600, MilliporeSigma, Burlington, MA), Olig2 (EPR2673, 1:100, Abcam, Cambridge, MA).

Tumor samples: Tumor samples were collected and reviewed by a pathologist to assure a minimum tumor percentage of 30%. Formalin-fixed paraffin embedded tissue (MNGT-1) or frozen tissue (MNGT-2) was extracted for DNA and RNA using standard protocols. Tumor only was tested. No paired normal tissue was available for comparison or filtration of germline variants.

Genetic evaluation: Genomic alterations were assessed using the CHOP Comprehensive Solid Tumor Panel. Fusion genes were measured by targeted RNA-seq using anchored multiplex PCR with custom designed primers (ArcherDx, Boulder, CO). Full exonic and select intronic/promotor sequence of 237 cancer genes were evaluated by next generation sequencing. Regions of interest were captured using SureSelect^{QTX} target enrichment technology (Agilent Technologies, Santa Clara, CA). All genes included

on the fusion and DNA based NGS sequencing panel are listed in Supplemental Table 4. Sequencing was performed on Illumina MiSeq or HiSeq (San Diego, CA). Sequencing data were processed using the homebrew software ConcordS v1 and NextGENe v2 NGS Analysis Software (Softgenetics, State College, PA). Briefly, sequence data were aligned to the hg19 (Grch37) reference genome using Novoalign and variants were called by various online tools, such as freebayes and VarScan. Variants were then processed and annotated using various databases including COSMIC (http://cancer.sanger.ac.uk/cosmic), The Cancer Genome Atlas (https://cancergenome.nih.gov/), ExAC (http://exac.broadinstitute.org), etc. The analytic sensitivity of the DNA sequencing portion of the assay was validated to 0.05 variant allele fraction (5% VAF). Variant interpretation was performed according to AMP/ASCO/CAP standards and guidelines for somatic variant interpretation and reporting (Supplemental Table 2) [7]. Copy number analysis from NGS was performed using NextGENe software (SoftGenetics, State College, PA).

Genetic Confirmatory Testing: *FGFR2-INA* fusion was confirmed by nested PCR amplification and Sanger sequencing of cDNA transcripts (see Supplemental Table 5). A blood sample from the patient with MNGT-2 was obtained and the *ATM* variant identified in the patient's tumor was also detected in the blood sample by targeted Sanger sequencing, confirming a diagnosis of ataxia-telangiectasia.

Vector Construction and Generation of Stable Cell Lines: FGFR2-INA constructs were synthesized as Gateway compatible entry clones. Sub-cloning was done to integrate FGFR2-INA into Gateway-compatible N-MYC-tagged pMXs-Puro Retroviral Vector (Cell Biolabs). Vector control was empty N-MYC-tagged pMXs-Puro Retroviral Vector. NIH3T3 and early-passage PMAs were transduced using infection protocol previously described [5,1]. Anti-MYC antibody (Invitrogen R951-25, 1:5000) was used to detect tagged fusion protein.

Western Blot Analysis: FGFR2-INA expressing NIH3T3 and PMAs were processed for western blot (WB) analysis as described previously [5]. For MAPK and PI3K/mTOR pathway analysis, pMEK (#9154), MEK (#4694), pERK (#4370), ERK (#4695), pAKT Ser473 (#4060), pAKT Thr308 (#4056), AKT (#2920), pS6 (#2215), S6 (#2317), and pFGFR(#3471) antibodies from Cell Signaling were used.

Soft-Agar Cellular Transformation Assays: Anchorage-independent growth in the presence or absence of inhibitors was assessed as previously described [1,5].

Co-immuoprecipitation Assays: To assess the dimerization potential of FGFR2-INA fusion, we tested homodimerization using myc- and flag-tagged constructs of FGFR2-INA and vector control in HEK293 cells that demonstrate high transfection efficiency. Following co-transfection, we performed immunoprecipitation (IP) of myc-tagged FGFR2-INA or control with a Myc antibody. If FGFR2-INA fusion could interact with itself and form homodimers, we expected to see pull down of flag-tagged FGFR2-INA but not control. The co-IP protocol used is as described previously [5].

Cellular drug assays: The MEK inhibitor, trametinib was provided by GlaxoSmithKline and LY3009120 (Eli Lilly, Indianapolis, IN, USA) and the mTOR inhibitor, everolimus was purchased from LC Laboratories, Woburn, MA, USA. All listed inhibitors were dissolved in dimethyl sulfoxide and stored at − 20 °C. Cells were plated at 1 × 106 cells/ml and serum-starved, followed by exposure to indicated drug concentrations for 1 h.

SUPPLEMENTAL FIGURE LEGENDS:

Supplementary Figure 1 (Online Resource 1). FGFR2-INA fusion protein expressed in heterologous cell models. Westerns showing stable expression of Myc-tagged vector control and FGFR2-INA (arrows) in NIH3T3 and p53-null mouse astrocyte cells (PMAs) using anti-Myc tag antibody. Additional blot showing non-specific band in 3 PMA lines including vector control, FGFR2-INA and QKI-RAF1 (previously published cell line [5]).

Supplementary Figure 2 (Online Resource 1). FGFR2-INA fusion driven signaling and oncogenicity are partially targeted by single agent treatment with MEK inhibitor, trametinib or mTOR inhibitor, everolimus. a. Western blot showing effect of treatment with single agents on NIH 3T3 cells expressing FGFR2-INA. **b.** Soft agar assays showing partial suppression of FGFR2-INA driven oncogenic growth in NIH 3T3 cells.

SUPPLEMENTAL TABLES (Online Resource 3)

Supplemental Table 1. Summary of the clinical, radiographic, histopathologic, and *FGFR2-INA* fusion in MNGT.

Supplemental Table 2. Single nucleotide variants identified in two MNGT tumors.
Abbreviation: VAF = Variant allele fraction, N/A = not applicable, SNV = single nucleotide variant
^Genomic position based on genome build hg19.
*Based on Li et al. 2017 JMD [7] except for ATM variant which was confirmed germline and is classified based on ACMG Criteria (Richards et al. 2015 PMID: 25741868)

Supplemental Table 3. Copy number variants identified in MNGT-2.
Note: No copy number changes were detected in MNGT-1
Abbreviations: WC- whole chromosome; cnLOH- copy neutral loss of heterozygosity

Supplemental Table 4. Targeted RNA-seq fusion panel genes and 237 cancer related genes sequenced by NGS.

Supplemental Table 5. PCR Primers for *FGFR-INA* fusion confirmation.
